# Evolving Mutational Buildup in HIV-1 Protease Shifts Conformational Dynamics to Gain Drug Resistance

**DOI:** 10.1021/acs.jcim.3c00535

**Published:** 2023-06-07

**Authors:** Michael Souffrant, Xin-Qiu Yao, Donald Hamelberg

**Affiliations:** † Department of Chemistry, Georgia State University, Atlanta, Georgia 30302-3965, United States; ‡ Center for Diagnostics and Therapeutics, Georgia State University, Atlanta, Georgia 30302-3965, United States

## Abstract

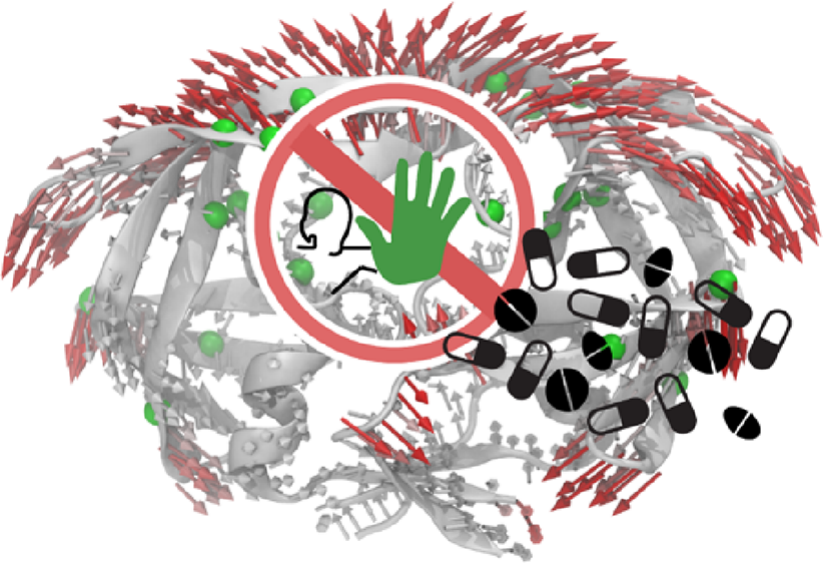

Drug resistance in antiviral treatments is a serious public health problem. Viral proteins mutate very fast, giving them a way to escape drugs by lowering drug binding affinity but with compromised function. Human immunodeficiency virus type I (HIV-1) protease, a critical antiretroviral therapeutic target, represents a model for such viral regulation under inhibition. Drug inhibitors of HIV-1 protease lose effectiveness as the protein evolves through several variants to become more resistant. However, the detailed mechanism of drug resistance in HIV-1 protease is still unclear. Here, we test the hypothesis that mutations throughout the protease alter the protein conformational ensemble to weaken protein–inhibitor binding, resulting in an inefficient protease but still viable virus. Comparing conformational ensembles between variants and the wild type helps detect these function-related dynamical changes. All analyses of over 30 μs simulations converge to the conclusion that conformational dynamics of more drug-resistant variants are more different from that of the wild type. Distinct roles of mutations during viral evolution are discussed, including a mutation predominantly contributing to the increase of drug resistance and a mutation that is responsible (synergistically) for restoring catalytic efficiency. Drug resistance is mainly due to altered flap dynamics that hinder the access to the active site. The mutant variant showing the highest drug resistance has the most ″collapsed″ active-site pocket and hence the largest magnitude of hindrance of drug binding. An enhanced difference contact network community analysis is applied to understand allosteric communications. The method summarizes multiple conformational ensembles in one community network and can be used in future studies to detect function-related dynamics in proteins.

## Introduction

Proteins are dynamic and malleable, undergoing spontaneous and induced intrinsic conformational motions under certain thermodynamic conditions. The conformational dynamics of a protein can be modulated by external stimuli (e.g., transient protein–protein interactions, small-molecule ligand binding, etc.) and modifications (e.g., posttranslational modifications, mutations, etc.), which together create what we term a perturbation. From a statistical mechanical point of view, perturbation modifies the energy landscape of the protein system, which in turn causes a shift of the conformational distribution over the landscape (i.e., the conformational ensemble) that eventually may lead to altered function. The dynamical changes can be both local and nonlocal; nonlocal changes connect multiple different compartments of the protein structure and provide the basis for the propagation of signals between distal sites (hence, explaining the allosteric effect). A critical step to detect these function-related conformational dynamical changes is to compare different conformational ensembles generated under different conditions,^1−7^ such as those belonging to the wild type (WT) and mutant variants of the protein. Specifically, comparing ensembles due to sequence variations provides a way to investigate the functional evolution of a protein family.^8^

**Figure 1:**
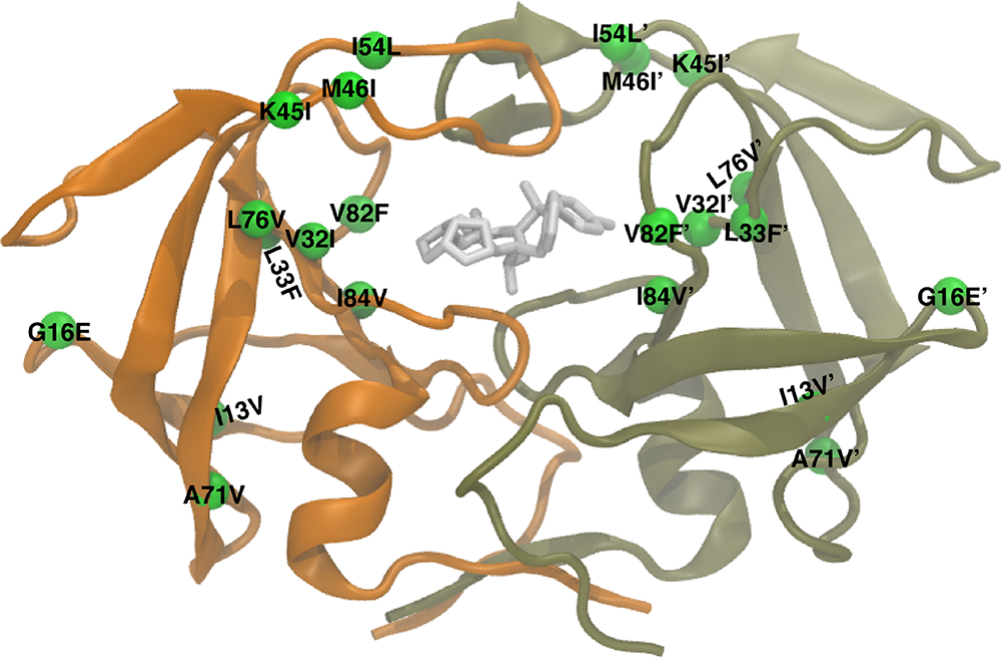
Structural representation of the homodimeric HIV-1 protease in complex with darunavir. Chains A and B are shown as cartoon colored in orange and tan, respectively. Mutation sites are shown as green beads. Darunavir, in complex with HIV-1 protease, is shown as white sticks. The studied mutations are derived from previous experimental work.^17^

HIV-1 protease, a viral enzyme encoded by the human immunodeficiency virus (HIV) type 1 genome, accumulates mutations under the pressure of antiretroviral therapy (Figure 1).^9,10^ The enzyme is a homodimer with two flaps covering the active site for regulation. This protease is responsible for the maturation of HIV and is capable of maintaining viable activities upon numerous mutations throughout its sequence.^11^ Under inhibition, the enzyme develops resistance to drug binding through many variants with different sets of mutations, while keeping adequate function, for the virus to survive. An example is the development of resistance against the current FDA-approved HIV-1 protease drug or inhibitor, darunavir (DRV).^12^ The drug inhibits the HIV-1 protease by occupying the active-site pocket,^13^ hindering catalysis. Because DRV is a potent inhibitor of the wild-type protease,^14−16^ it requires the accumulation of several mutations for the enzyme to gain significant resistance.

HIV-1 protease develops drug resistance against DRV by evolving through various mutant variants.^17^ However, how the buildup of mutations leads to drug resistance is not well understood. The phenomenon is expected to be mediated by a shift in the conformational ensemble since many of the mutations are distal to the active site. In addition, the potential pathways to resist inhibition from many of the mutation sites to the active site are unclear. The tug of war between producing a viable ensemble under inhibition and having sufficient catalytic efficiency as the enzyme mutates is not well understood either. Previous molecular dynamics(MD) simulations on HIV-1 protease^17−24^ sampled the protein at the nanosecond scale to elucidate the enzyme dynamic properties and improve drug design to oppose the events of drug-resistant mutations. Sampling at the microsecond scale could reveal larger-amplitude conformational changes, which is essential for understanding how the enzyme develops DRV resistance through mutations.

**Figure 2:**
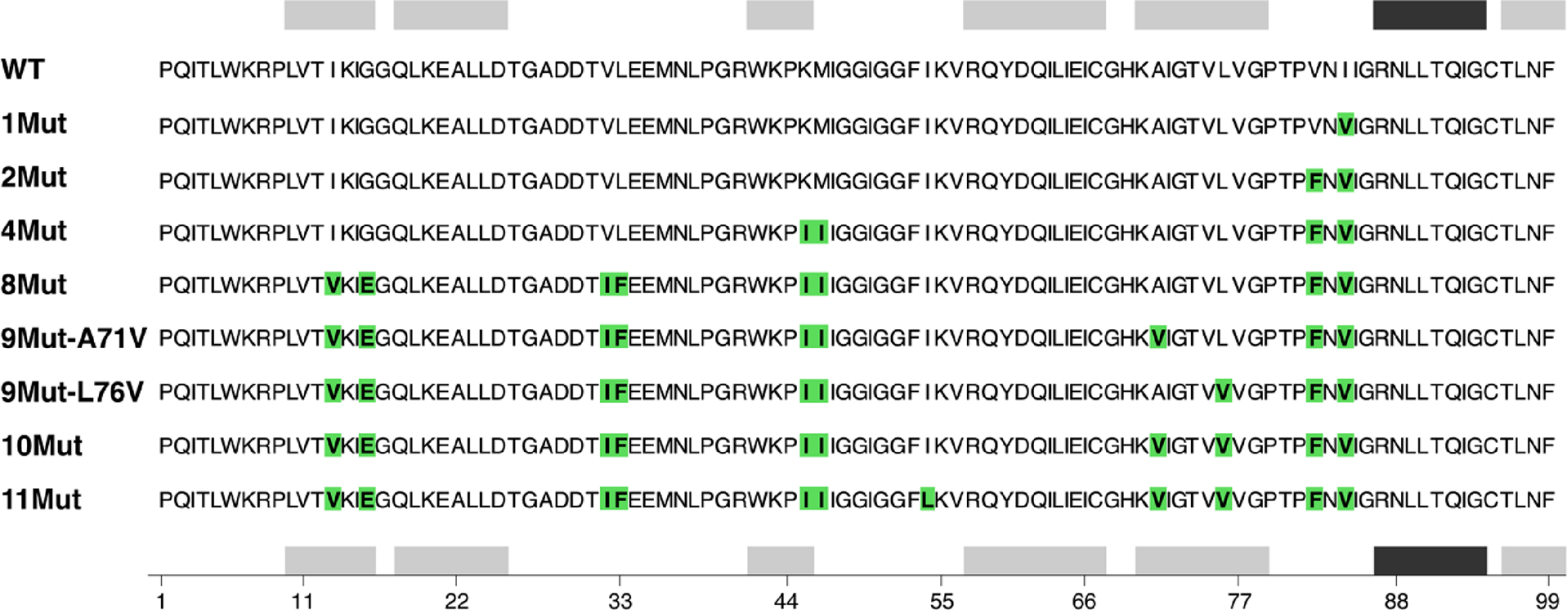
Amino acid sequence alignment of the HIV-1 protease variants. The sequence for one monomer of the homodimer is shown. Mutation sites are colored in green, whereas α-helices and β-strands are shown as black and gray rectangles, respectively.

In this study, we sample the dynamics of multiple HIV-1 protease variants (Figure 2), including the wild type (i.e., the variant susceptible to DRV) and several DRV-resistant mutant variants, free in solution using multiple microsecond-long MD simulations. We compare the dynamical changes of the mutant variants with respect to the wild-type enzyme. Our work reveals statistical correlations between flap dynamics and the ability of drug resistance of the enzyme. Atomic motions of the flaps are critical for both drug and substrate to access the active-site and, hence, would impact the enzyme activity. Due to the introduction of drugs, HIV-1 protease evolves by adjusting the conformational dynamics through mutations to find a balance between catalytic viability and drug resistance.

## Methods

### Molecular Dynamics

In general, molecular dynamics (MD) simulations to generate the trajectories of nine systems, started from the X-ray crystal structure of HIV-1 protease (PDB: 1T3R),^12^ were carried out for multiple microseconds. Each system was simulated free in solution, unbound from darunavir. Mutant variants, studied in previous experimental work,^17^ were modeled based on the crystallographic structure of wild-type HIV-1 protease (Figure 2). The amino acid names of the wild-type enzyme were changed to match the mutant in the PDB, and mutation site side chain atoms were removed. Using LEAP module in AmberTools, missing side-chain atoms were added back in accordance with the new sequence. Simulations were carried out using Amber16 programs.^25^ The Amber ff14SB,^26^ a modified version of Cornell et al.,^27^ was used for the simulations. Each system was solvated with TIP3P explicit water in an octahedron box with each face 10 Å away from the enzyme’s surface^28,29^ and then neutralized using counter ions (Na^+^ or Cl^–^)^30^ accordingly. Aspartate 25, the catalytic residue of chain A, was protonated to stabilize the repulsive interaction with the mirrored (unprotonated) aspartate from chain B. Systems were simulated using a 2 fs time step at a temperature of 300 K and a pressure of 1 bar. The temperature was maintained using the Langevin thermostat (collision frequency 1.0 ps^–1^), whereas the pressure was based on a Monte Carlo barostat (with a coupling constant of 1.0 ps). Bonds with hydrogen atoms were constrained using the SHAKE algorithm.^31^ The particle mesh Ewald (PME) summation method was used for long-range electrostatic interactions.^32^ Short-range non-bonded interactions were set at a 9 Å cutoff. All systems were energy minimized for 15,000 steps, in which case harmonic restraints with a force constant of 500 kcal/mol·Å^2^ were used for the first 5000 steps on the protein and the second 5000 steps on the solvent/ions. The remaining 5000 steps of minimization were devoid of any restraints. For each round of the 5000-step energy minimization, 2000 steps of steepest descent followed by 3000 steps of conjugate gradient were performed. Systems were then equilibrated for a total of 3.5 ns with reducing harmonic restraints from 500 to 0 kcal/mol·Å^2^ on the protein. In production, systems were simulated for at least 2.1 μs: 1Mut (2.1 μs), 2Mut (2.1 μs), 4Mut (4.1 μs), 8Mut (4.1 μs), 9Mut-A71V (2.1 μs), 9Mut-L76V (4.1 μs), 10Mut (2.1 μs), 11Mut (5 μs), and WT (5 μs). These accumulate a total of 30.7 μs simulation data. The entirety of each trajectory was used to analyze different conformational characteristics of HIV-1 protease upon its proximal and allosteric (with respect to the active site) mutations. Root-mean-square fluctuation (RMSF) calculations, principal component analysis (PCA), distance distribution analysis of flap residues, and residue–residue contact statistics were then carried out for comparative probing of the MD trajectories. RMSF was calculated based on the backbone atoms (N, Cα, C, and O) of the simulations, through CPPTRAJ applications. Prior to RMSF calculations, conformations were superimposed onto the first frame of the simulation based on backbone atoms. RMSF was averaged within each residue and over the simulation. ΔRMSF of the mutant variants was evaluated in percent change with respect to WT.

### Principal Component Analysis

Using backbone atoms (N, Cα, C, and O), frames from the trajectories were superimposed on the first frame of the WT simulation. Principal component analysis was then carried out through CPPTRAJ of AmberTools on the Cartesian coordinates of the backbone atoms obtained from the trajectories.^33^ The variance–covariance matrix was calculated to describe the correlated internal motion of the enzyme from the trajectories. Then, the matrix was diagonalized to produce the eigenvectors and their respective eigenvalues. The eigenvectors were projected back onto the trajectories as principal components (PCs). As such, the principal component with the largest eigenvalue corresponded to the greatest variance of conformational sampling in the trajectories. PC1 and PC2, the top two principal components, were projected on the WT on a 2-D PC plot using the ggpplot2 R package.^34^ Conformational motions of the PC1 eigenvector were visualized using VMD.^35^ Conformational distributions of the HIV-1 protease mutant variants along PC1 were evaluated and were compared to WT.

### Contact Network Analysis

Contact statistics were calculated as previously described in Doshi et al.^36^ When any pair of heavy atoms, from different residues (*i* to *i* + *n*, *n* ≥ 3), are within 4.5 Å of each other, a contact is considered to be formed. The probability of contact formation, *p_c_*, during a simulation was calculated, in which case, contacts formed less than 10% throughout each trajectory were not considered, as those contacts either never formed or occurred at a probability too low for significant comparisons between WT and the mutant variants (*p_c_* close to 0). The difference contact network analysis (dCNA)^37^ was performed by using contacts with high probability of formations (*p_c_* ≥ 0.9), from all trajectories to detect communities via Girvan–Newman’s algorithm^38^ and the network modularity analysis.^39^ In the following analysis, Pearson’s correlation coefficient was calculated to evaluate the correlation of the net residue–residue contact probabilities of formation in between communities with the logarithm of the inhibition constant (*K*_i_)^17,18^ values of the mutant variants and WT. The enzyme inhibition constant, *K*_i_, of each variant against DRV was measured from previous experimental work: WT (*K*_i_ = 0.005 nM), 1Mut (*K*_i_ = 0.026 nM), 2Mut (*K*_i_ = 0.23 nM), 4Mut (*K*_i_ = 0.42 nM), 8Mut (*K*_i_ = 12.8 nM), 9Mut-A71V (*K*_i_ = 23.2 nM), 9Mut-L76V (*K*_i_ = 172.7 nM), 10Mut (*K*_i_ = 156.4 nM), and 11Mut (*K*_i_ = 759.2 nM).^17,18^ The residue communities and correlation analysis was performed using igraph^40^ and Bio3D^41−43^ R packages.

## Results and Discussion

### Contact Dynamics of the Mutant Variants Become Less Correlated to That of Wild-Type HIV-1 Protease upon Increase of Drug Resistance

As noted from previous studies,^44,45^ certain mutations in HIV-1 protease cause an increase of drug resistance. We assume that mutations shift the conformational ensemble, which modifies protein–drug interactions. Here, we use residue–residue contact statistics of HIV-1 protease to characterize and compare the perturbed ensembles caused by the mutations. As the sequence varies, new contacts are generated and some existing contacts disappear; hence, contact analysis provides a way to examine divergence of the mutant variants, with respect to dynamical characteristics and function, from the wild type.

**Figure 3:**
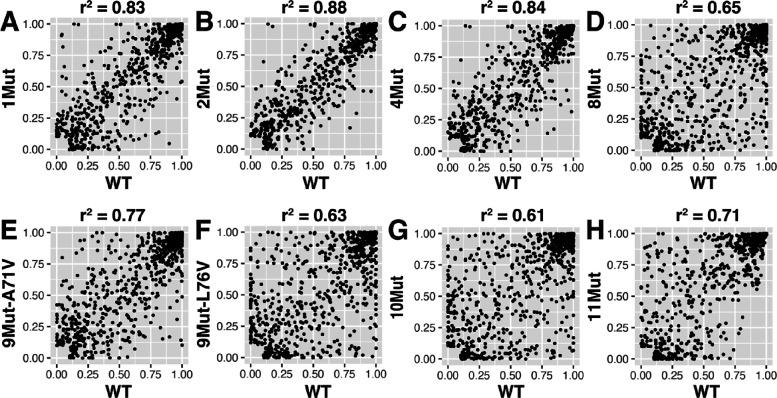
Comparison of residue–residue contact statistics between the mutant variants and WT. Each residue–residue contact is represented by a point on the plot with its ordinate and abscissa corresponding to the contact probabilities, *p_c_*, for the mutant variant and WT, respectively. Contacts formed less than 10% of each trajectory are not included for this analysis. The coefficient of determination (*r*^2^) is included for each plot to quantify the strength of the correlations.

Comparing residue–residue contact statistics of the mutant variants with the wild-type enzyme shows a loss in contact correlation as the number of mutations (and hence drug resistance) increases (Figure 3). Such findings suggest new contact dynamics (i.e., contact formation or breakage) from the mutant variants, not seen in WT (or vice versa), as HIV-1 protease becomes more resistant to DRV. Interestingly, the contact correlation of 9Mut-A71V with WT (Figure 3E) is more comparable to the correlation results from 1Mut, 2Mut, and 4Mut (Figure 3A–C), whereas the contact correlation of 9Mut-L76V (Figure 3F) resembles that of systems with more mutations (i.e., 8Mut and 10Mut) (Figure 3D,G). This distinction in contact correlation with WT between 9Mut-A71V and 9Mut-L76V (named hereafter as “engineered mutants”) suggests that each mutation causes different conformational changes on the enzyme upon resisting the drug. The relative ranking based on contact correlation with the WT between the two engineered mutants is consistent with drug resistance abilities of the mutants: Previous experiments have shown that 9Mut-L76V has a higher drug resistance (*K*_i_ = 172.7 nM), at a level similar to 10Mut, than 9Mut-A71V (*K*_i_ = 23.2 nM).^17^ However, we note that 9Mut-A71V is categorized as a high drug-resistant variant according to the inhibition constant, even though the contact correlation with the WT of the mutant shifts toward those of low drug-resistant variants.

Previous findings from the double mutant cycle analysis^17^ showed the interdependency of A71V and L76V to offset drug inhibition as the enzyme mutates from 8Mut enroute to 10Mut. Specifically, the sum of increases of drug binding free energy (i.e., drug resistance ability) gained by individual 9Mut mutations is slightly higher than that gained by the double mutation, suggesting that the synergy between the two mutations has a moderate negative effect on drug resistance. Furthermore, viruses with 9Mut-A71V and 9Mut-L76V variants of the enzyme were not efficiently replicated to be detected in viral passaging.^17^ Both mutants were shown to have significantly low efficiency (i.e., 171-fold lower than WT).^17^ This suggests that the interaction between the two mutations is responsible for the recovery of catalytic efficiency from 9Mut to 10Mut, at a small cost of drug resistance. The high drug resistance of 9Mut-L76V indicates that L76V alone contributes to the large increase of *K*_i_ from 8Mut to 10Mut. The addition of A71V mutation recoups the loss in catalytic activity of 9Mut-L76V to produce a more viable variant in viral passaging as 10Mut. Previous findings^46,47^ on A71V also suggest that the mutation has compensatory properties on the lost enzyme activity caused by earlier mutations.

In summary, our analysis reveals a relationship between residue–residue contact dynamics and the magnitude of drug resistance of HIV-1 protease. The overall pattern of contact probabilities is more different from that of the WT for the more drug-resistant mutants, suggesting the shift of contact dynamics through mutations that renders the enzyme to be less catalytically competent but more resistant to DRV. The 9Mut-L76V mutant is predicted to be primarily responsible for the enhanced drug resistance from 8Mut to 10Mut. Accordingly, A71V is assumed to be a restoring mutation because 9Mut-L76V is much less efficient than 10Mut.

### Accumulation of Mutations Alters the Conformational Dynamics of the Flaps

HIV-1 protease cleaves the Gag and Pol polyproteins at different sites for viral assembly and maturation.^48^ The two flaps enclose the active-site binding pocket of the enzyme. The flaps regulate catalytic function by allowing or blocking access to the active-site cavity. Previous distance analysis between I50 Cα atoms of the two monomers was evaluated to monitor flap conformations of ligand-free HIV-1 protease at different temperatures of molecular dynamics simulations.^49^ The study indicated that the flaps may exist in different ensembles of conformations (semi-open, open, and curled), which may influence entrance to the active site during catalysis. Therefore, inspecting the distance between the flaps provides information on flap dynamics and the cleaving properties of the enzyme throughout the different variants.

**Figure 4:**
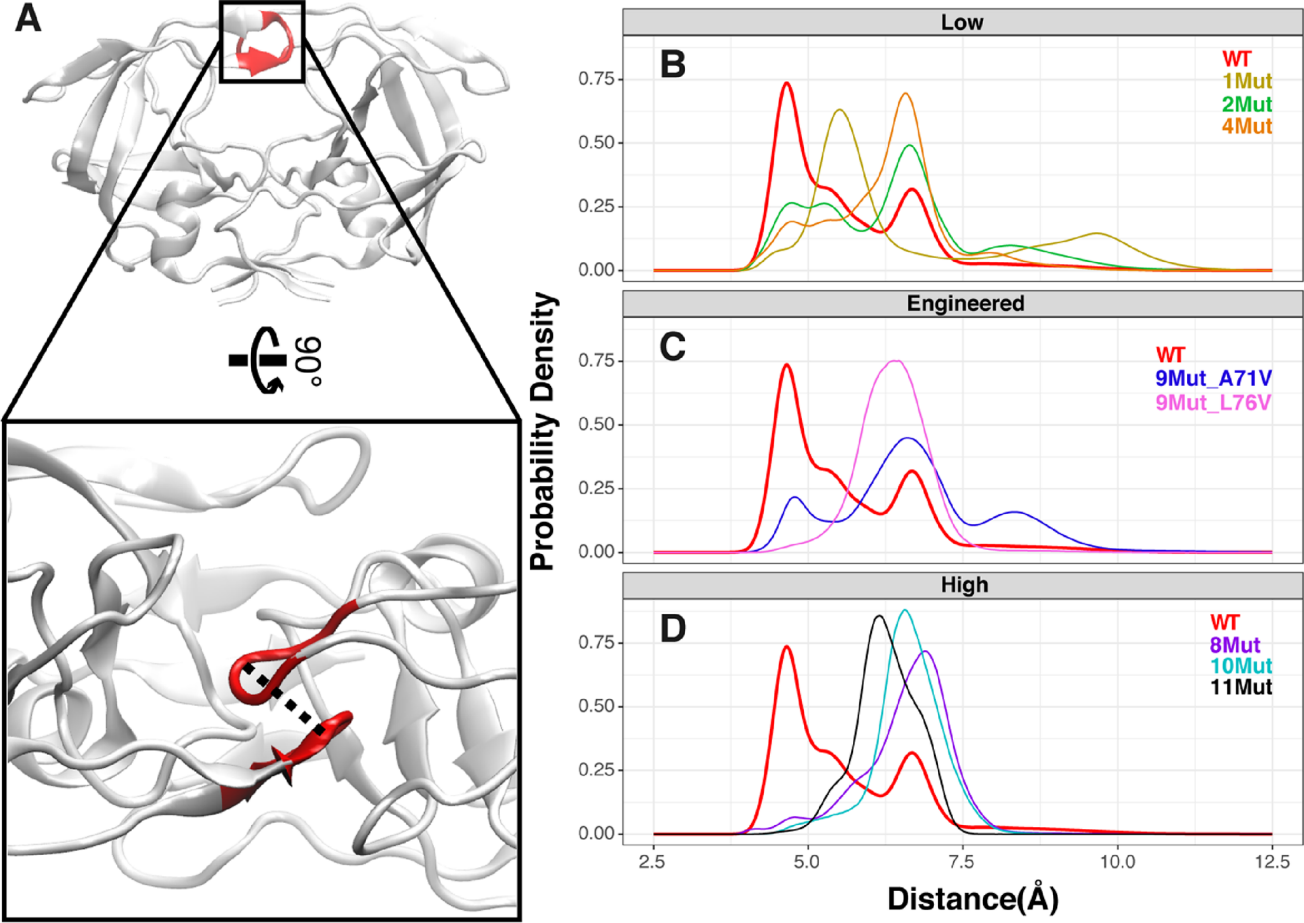
Comparative distance analyses of homodimeric flaps between WT and mutant variants. (A) Structural representation of the wild-type HIV-1 protease. Flap tips (49–52; 49′–52′) are shown in red with the enzyme structure rotated at a 90° angle to show the probed distance between flaps. The distance distribution for WT (red) is shown in panels (B)–(D) in relation to the mutant variants. (B) Distance distributions of WT and the low-resistance mutant variants to DRV: 1Mut (yellow), 2Mut (green), and 4Mut (orange). (C) Distance distributions of WT and the engineered mutant variants: 9Mut-A71V (blue) and 9Mut-L76V (violet). (D) Distance distributions of the WT and the high-resistance mutant variants to DRV: 8Mut (purple), 10Mut (cyan), and 11Mut (black).

The distance between the center of mass of the flap tips (49–52; 49′–52′) is probed for each trajectory, in which case distance distributions are compared in relation to WT (Figure 4). The distribution of the wild-type enzyme shows two major distances sampled between the flaps at ∼4 and ∼7 Å, with the shorter distance having a higher probability density. Upon mutation, however, the longer distance (i.e., ∼7 Å) is consistently sampled at a greater probability, indicating a shift in flap dynamics that correlates with drug resistance. Mutant variants with low resistance to DRV (i.e., 1Mut, 2Mut, and 4Mut) show higher flap flexibility compared to the WT as the enzyme samples different distances between the flap tips (Figure 4B). Flap plasticity potentially lowers the catalytic activity by altering access to the active-site cavity. The 1Mut, 2Mut, and 4Mut variants have similar *K*_m_ values to the WT but the *k*_cat_ values of the variants are 2- to 7-fold lower.^17^

For the engineered mutant variants, 9Mut-A71V samples a multiple-peak distance distribution similar to the low-resistance 1Mut, 2Mut, and 4Mut, whereas 9Mut-L76V shows a distribution with a single peak localized at ∼7 Å as in the high-resistance 8Mut, 10Mut, and 11Mut (Figure 4C). Such findings agree with the contact correlation results of the engineered mutant variants with WT, where the contact statistics for 9Mut-L76V is more similar to that of the high-resistance variants. This further supports the hypothesis that L76V is more responsible for the increase of drug resistance between the two engineered mutants during evolution in viral passaging of 8Mut enroute to 10Mut, whereas the addition of A71V is mainly to restore the catalytic efficiency for survival.

### Drug-Resistant Mutations Affect the Overall Conformational Dynamics of HIV-1 Protease

**Figure 5:**
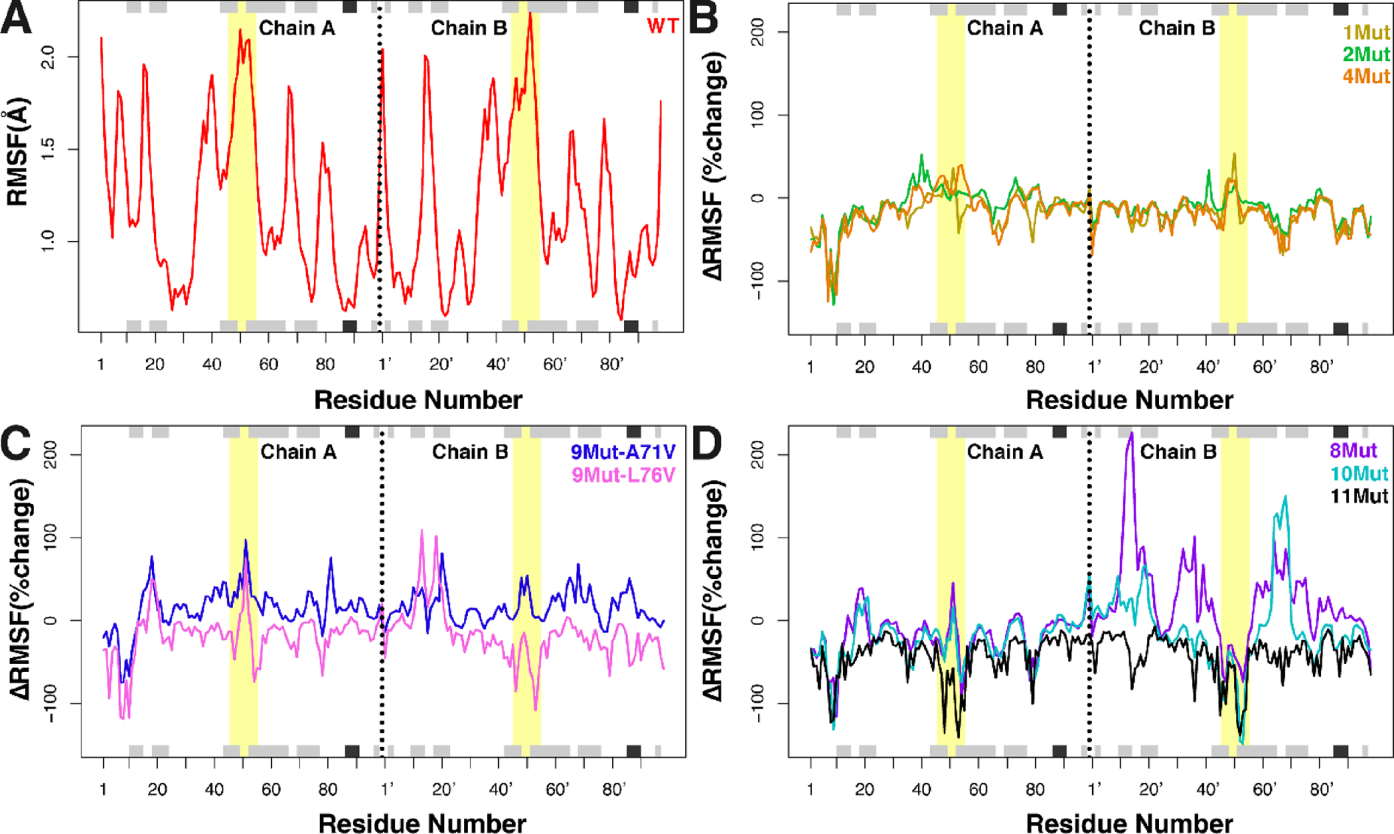
RMSF of the WT relative to mutant variants. (A) Residue-wise averaged RMSF of the wild-type HIV-1 protease based on the backbone atoms. (B–D) ΔRMSF of the mutant variants in percent change relative to (A) for 1Mut (yellow), 2Mut (green), 4Mut (orange), 9Mut-A71V(blue), 9Mut-L76V (violet), 8Mut (purple), 10Mut (cyan), and 11Mut (black). Chains A and B are labeled accordingly to represent the monomers of the structure. Flap regions (residues 45–55; 45′–55′) are shown in yellow as the highlighted areas of each plot. The black and gray rectangles on the top and bottom of each plot represent α-helices and β-strands, respectively.

Residue-wise averaged RMSF of the WT depicts significant fluctuations in many regions of the structure (Figure 5A). The flap regions (residues 45–55; 45′–55′) have similar high backbone fluctuations in both chains A and B of the homodimer. Differential fluctuations (ΔRMSF) of the mutant variants are calculated in percent change with respect to WT (Figure 5B–D). As the number of mutations increases, ΔRMSF of the variants becomes more noticeable. This suggests an ensemble that is experiencing more changes in backbone fluctuations, with the accumulation of different mutations, compared to its original point of reference (i.e., WT).

The mutant variants with low resistance to inhibition show small changes in backbone fluctuations when compared to the WT (Figure 5B), including the ΔRMSF of their flap regions. This indicates an enzyme that initially loses catalytic efficiency and gains drug resistance from different mutations, without deviating too much from the WT in its conformational fluctuations. Compared to the engineered mutant variants (Figure 5C), the ΔRMSF of 9Mut-A71V is closer to zero than the ΔRMSF of 9Mut-L76V. Hence, the 9Mut-A71V mutant variant generates backbone fluctuations that are more akin to the wild-type enzyme than that of 9Mut-L76V. This supports the previous conclusion that 9Mut-L76V is responsible for gaining drug resistance, whereas the addition of A71V restores catalytic efficiency during the transition from 8Mut to 10Mut. Interestingly, the ΔRMSF of 11Mut (Figure 5D) shows a negative percent change relative to the WT throughout the protein, which differs from the other variants. This supports the previous suggestion that 11Mut gains the highest resistance by impeding access to its catalytic pocket the most, compared to other mutant variants and WT.

### Mutations Cause “Collapse” of the Active-Site Cavity to Prevent Drug Inhibition

Previous work^20^ on HIV-1 protease suggested a correlation between the flaps’ conformational dynamics and reorganization of residues in the active site. Furthermore, the preorganization of active site residues was considered to be a rate-limiting factor in catalysis. Other studies suggested the occurrence of polymorphisms,^48^ including compensatory mutations,^50,51^ in Gag polyprotein cleaving sites in response to the change in catalysis of the enzyme. This suggests a collective effort of different viral proteins to find the balance between drug resistance and catalytic efficiency for survival. To further inspect how the viral mutations impact flap conformations to lower catalytic efficiency while making the enzyme more resistant as a drug target, we performed principal component analysis. PCA focuses on the dominant enzyme motion embedded in a large conformational space, and it identifies different conformational states by projecting conformations onto their top eigenvectors (i.e., principal component or PC). PCA reduces the dimension of the conformational space of HIV-1 protease, which makes it easier to elucidate the variation perturbation of the conformational ensembles caused by the mutations.

**Figure 6:**
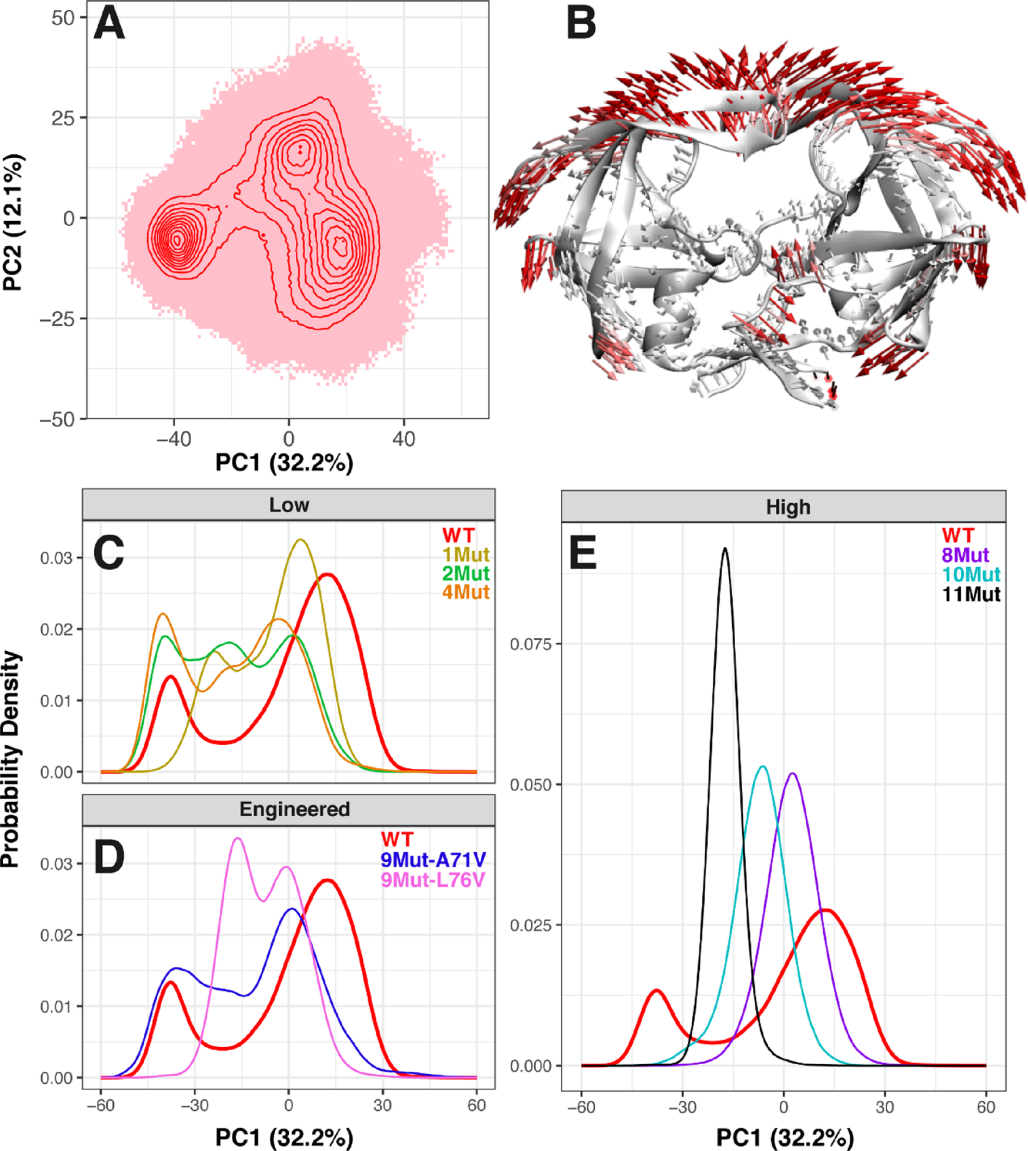
PCA of wild-type HIV-1 protease compared to the mutant variants. (A) Conformer plot projected onto the PC1–PC2 subspace of the wild-type enzyme. Simulation-generated conformations are displayed as red shaded areas, with contour lines showing the most populated conformational states. The axis label indicates the percentage of the total structural variance captured by each PC. (B) PC1 motion mapped on the structure of the wild-type HIV-1 protease. Arrows indicate the collective motion associated with PC1 and are color coded by their lengths (from white to red with the increase of length). Lengths of arrows are scaled by the weights of corresponding atoms in the PC, and arrows shorter than 1 Å are removed for clarity. (C–E) PC1 distribution of WT (red) relative to the mutant variants. (C) PC1 distributions of WT and the mutant variants with low resistance to DRV: 1Mut (yellow), 2Mut (green), and 4Mut (orange). (D) PC1 distributions of WT and the engineered mutant variants: 9Mut-A71V (blue) and 9Mut-L76V (violet). (E) PC1 distributions of WT and the mutant variants with high resistance to DRV: 8Mut (purple), 10Mut (cyan), and 11Mut (black).

Based on the scree plot of the WT PCA analysis (Figure S1), the top two principal components (i.e., PC1 and PC2) represent ∼44.3% of the total structural variance of the conformational motions of the enzyme. As such, Figure 6A shows the PC1 and PC2 plot of the governing conformational states of wild-type HIV-1 protease throughout the trajectory. The plot contains a total of three separate peaks (with respect to the density of conformational sampling), displayed by the contour lines, indicating different populated conformational states. The structural mapping of WT PCA (Figure 6B) mirrors the range of conformations defining the peaks along PC1, the highest principal component. The arrows indicate a coordinated outward motion of the flaps, with the entirety of the structure going from −40 to 40 Å (at PC1). Interestingly, most long arrows (representing the atoms that hold the most weight in the PC) are located on the outer surface of the structure, whereas arrows within the catalytic site are short as they hold less weight along PC1. The result suggests that the dominant motion of the wild-type enzyme is largely defined by flap dynamics. “Untwisted” (i.e., PC1 at 40 Å) and “twisted” (i.e., PC1 at −40 Å) flaps are possibly related to the catalytic activity.

The probability density of conformations from WT along PC1 (Figure 6C–E) denotes two major peaks, which echoes the two flap-(un)twisted states of the enzyme. Once projected on the WT PC vectors, the conformational probability density distributions of the PC1 of the mutant variants, however, are different from that of the WT. Mutant variants with low drug resistance show probability densities with either multiple (>2) peaks or two peaks of shifted positions (compared to WT), with the population of conformations between the twisted and untwisted states having a higher probability density (∼0.015), indicating a lower energy barrier, than that in WT (∼0.005). Hence, at the beginning of evolution (i.e., 1Mut, 2Mut, and 4Mut), the flaps become more flexible and easier to transition between the (un)twisted states, which may impact catalytic function and inhibition through modulating the dynamics of the active-site cavity. The enzyme reduces flap stability to weaken drug binding interactions. This in turn causes a loss in catalytic efficiency since both substrate binding and cleaving properties of the enzyme are also altered. As mutations accumulate (i.e., 8Mut, 10Mut, and 11Mut), the enzyme generates a single-peak probability density along PC1, indicating an optimization of the conformational ensemble for inhibitor resistance (Figure 6E). All the three mutants populate conformations between the twisted and untwisted states, with the more drug-resistant variant populating more closely to the twisted state. The result suggests a “collapse” of the flaps into the binding pocket in these mutant variants, blocking the cavity of the catalytic site. The 11Mut variant represents the most “collapsed” variant, which may explain the highest drug-resistant ability of this enzyme. Compared to the WT, the two flaps of 11Mut are more limited in their range of conformation, rendering the structure dynamically defective. The 11Mut variant is 15-fold lower in enzyme turnover number (i.e., *k*_cat_) and 13-fold lower in catalytic efficiency relative to the WT.^17^

Both engineered mutant variants show two peaks in their corresponding PC1 probability densities (Figure 6D). The PC1 distribution for 9Mut-A71V includes conformational states from the “twisted” and “untwisted” peaks of WT, a characteristic shown from the mutant variants with low inhibition constants (i.e., 1Mut, 2Mut, and 4Mut). The 9Mut-L76V variant comprises more selective conformational states, with PC1 probability peaks becoming more localized as shown in 8Mut, 10Mut, and 11Mut. Noticeably, 9Mut-L76V samples frequently around the probability density peak of 10Mut, which is not the case for 9Mut-A71V. Moreover, 9Mut-L76V has a lower transition barrier separating its two peaks (or wells with respect to free energy) in comparison to 9Mut-A71V. Likely, as 8Mut mutates to 9Mut-L76V, the enzyme generates a more feasible energy barrier to start sampling for the conformational states seen in 10Mut. Then, the additional A71V creates a synergistic impact with 9Mut-L76V to produce the single PC1 peak of 10Mut. This again suggests that the L76V mutation from 8Mut enroute to 10Mut contributes the most to the increase of drug resistance, whereas the addition of A71V restores the catalytic efficiency, in accordance with previous residue–residue contact statistics, ΔRMSF, and flap distance distributions analysis of the engineered mutant variants. Hence, our results from probing the conformational ensembles of the 9Mut-L76V and 9Mut-A71V versus the other mutant variants, including WT, converge to the same conclusion. The distinct roles of the two mutations during viral evolution from 8Mut to 10Mut suggest a structure undergoing changes to resist inhibition, while maintaining sufficient efficiency during catalysis.

### Contact Network Community Analysis Identifies Key Conformational Changes throughout the Protein That Are Correlated with Drug Resistance

As a homodimer, HIV-1 protease creates a catalytic pocket that is targeted for inhibition. The active site resembles a tunnel covered by two flaps. The flaps open and close upon substrate binding, thus regulating catalytic function through hydrolysis of the peptide bond. Moreover, many mutations from viral passaging to resist drug inhibition are far from the active site, suggesting long-range communications between different parts of the structure through inter-residue contacts, which is also proposed in previous findings^52,53^ as the “network hypothesis”. Hence, the difference contact network analysis (dCNA)^37^ is applied to analyze the contact changes through community networks, which generates a simpler representation of the interactions between and within monomers, including domains and/or subdomains of the enzyme. Instead of calculating intercommunity contact changes between two distinct conformational ensembles as previously described,^37^ here, we summarize multiple ensembles by calculating the correlation between intercommunity contacts and the logarithm of the inhibition constant (*K*_i_)^17^ across all the studied systems (i.e., residue communities and correlation analysis). This provides an efficient way to examine the impact of the allosteric mutations on the contact network in between communities and the meaningful conformational changes occurring within the enzyme as the enzyme becomes more resistant to drug inhibition.

**Figure 7:**
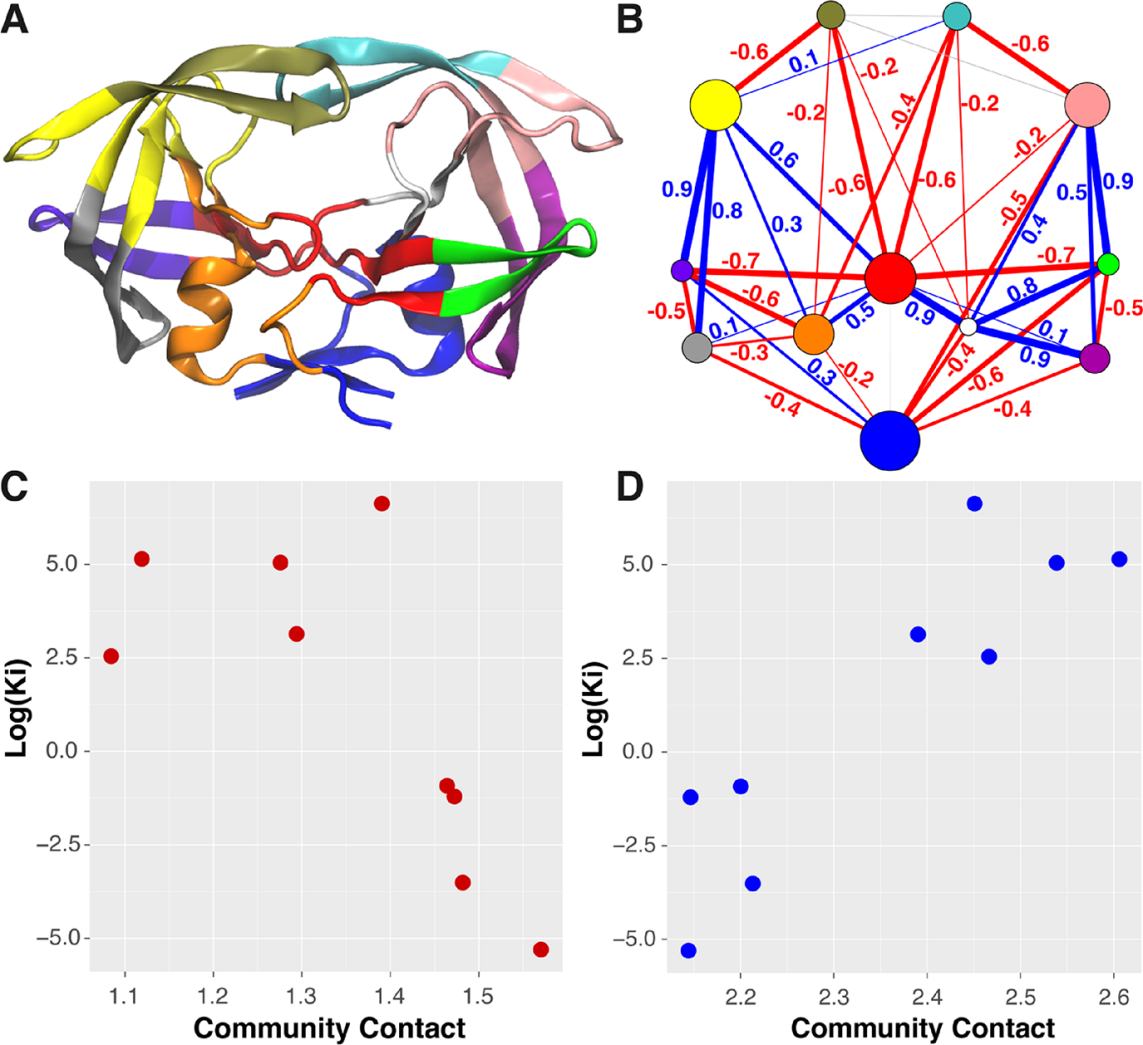
Residue communities and correlation analysis between community–community contacts and the drug resistance profile involving wild-type HIV-1 protease and the mutant variants. (A) Mapping of 12 consensus communities, indicated by different colors, onto the structure of HIV-1 protease. (B) Vertices represent the communities color coded the same as in panel (A). The more residues included within a community, the larger its corresponding vertex radius. Lines represent the correlation between the sum of residue–residue contact probabilities between two communities and the logarithm of the inhibition constant (*K*_i_) values for 9 mutant strains of HIV-1 protease from the WT through the mutations. Line width is scaled by the magnitude of Pearson correlation coefficient. Negative and positive correlations are shown as red and blue, respectively. Correlations with magnitude less than 0.1 are shown as gray. (C) Example of negative correlation associated with the line between the red and purple communities in panel (B). (D) Example of positive correlation associated with the line between the purple and yellow communities.

Twelve consensus communities are mapped on the HIV-1 protease structure as shown in Figure 7A (the number of communities is determined by maximizing the modularity as shown in Figure S2). Contact statistics results of HIV-1 protease are averaged over monomers to consider the symmetry of the homodimeric structure. However, the consensus community network derived from dCNA is not necessarily symmetric due to intermonomer interactions. This results in an overall asymmetric distribution of network edges that is quantified by the correlation between intercommunity contact statistics and the logarithm of the inhibition constant (*K*_i_). The flaps of chains A and B are colored as tan and cyan, respectively. The red community shared between the two monomers mostly represents the catalytic residues in the active-site binding pocket of the enzyme. An example of a negative correlation (ρ = −0.7) is shown in Figure 7C to probe the loss of residue–residue contacts between the red and purple communities as the enzyme becomes more resistant to DRV. On the contrary, Figure 7D represents an increase in residue–residue contacts between the purple and yellow communities as resistivity to DRV becomes more prevalent (ρ = 0.9). These plots, having a detailed depiction on the extent of correlation of residue–residue contacts with resistance, can help identify more meaningful conformational changes than the Pearson correlation coefficient that may be affected by outliers. For instance, the gray line between the cyan and tan communities (Figure 7B) is indicative of a correlation less than 0.1. However, the scatter plot of the line (Figure S3) shows an overall negative correlation except for the outlier represented by the 11Mut variant. As such, the flaps of the enzyme initially form less and less residue–residue contacts upon mutating from low to high DRV resistance, with a sacrifice of the efficiency. As the enzyme evolves to the 11Mut variant (the addition of the I54L mutation) however, residue–residue contacts are more frequently formed between the flaps, producing the outlier that affects the overall Pearson correlation coefficient to a magnitude of less than 0.1. Since access to the active-site pocket would be barred by the enhanced interaction between the flaps, the 11Mut enzyme becomes less prone to both substrate and inhibitor binding (resulting in compromised catalytic function and inhibition). It is worth noting that ligand proximity (i.e., substrate and inhibitor) could also influence flap conformations in 11Mut, by breaking the network of interactions in between the flaps and hence allowing access to the active site through an induced-fit mechanism. However, such events would not be inconsistent with the low catalytic efficiency (1.3 ± 0.4 μM^–1^ s^–1^) and the high inhibition constant (759.2 nM) of 11Mut^17,49^ because the mutant intrinsically prefers a “flaps-closed” conformation as revealed by our ligand-free simulation.

Mutations occur throughout the different vertices of the community network (Figure 7B) except for the blue community that is shared between the monomers. Yet, the blue community shows various interactions with other communities from the network that are correlated with the drug resistance profile, indicating an allosteric effect of mutations on drug binding. It is worth noting that not all interactions with the blue community are related to drug resistance. For example, the blue community also interacts with the red community because the two are spatially close, but the interaction has a correlation of less than 0.1 with the resistance profile (Figure 7A). The proximal mutations (i.e., I84V, V82I, and V32I) to the active-site cavity are included in the orange (I84V and V32I) and yellow (V82I) communities of chain A and the white community of chain B. All three communities form contacts with the red community that are positively correlated with the logarithm of the inhibition constants. In contrast, communities with distal (from the active site) mutations have interactions with the red community that are either positively or negatively correlated with the DRV resistivity. Interestingly, previous studies have found that mutations proximal to the active site are hypothesized to have impact on resistance by affecting binding, while distal mutations may be responsible for restoring catalytic function of the enzyme.^54−57^

In summary, our contact network community analysis describes conformational changes (represented by the shift of residue–residue contacts) between specific parts of the structure as the enzyme becomes more drug resistant. Active-site proximal mutations can directly affect drug and substrate binding to find a balance between drug resistance and catalytic efficiency. Distal (from the active site) mutations, on the other hand, may achieve the same target through allosteric effects, using the identified community network and associated conformational changes as the mechanism of communication between the distal and active sites. It is important to note that the direct effect of the buildup of mutations throughout the structure of HIV-1 protease is to alter enzyme dynamics, whereas the relation between mutations and *K*_i_ is essentially a correlation, which may not always hold in all systems.

## Conclusions

In this study, we generate conformational ensembles of the wild-type HIV-1 protease and its different mutant variants with increasing drug (i.e., DRV)-resistant abilities under the drug-free condition. We compare the distinct ensembles to describe the impact of allosteric and proximal (with respect to the active site) mutations on enzyme activity against inhibition. The variation of ensemble reflects evolution of the energy landscape of the viral enzyme from WT to drug-resistant mutants, which establishes a viable enzyme that samples conformational states with low affinity for inhibition. As such, analysis tools (i.e., contact statistics, flap distance analysis, RMSF, PCA, and dCNA-based residue community correlation analysis) provide more relevant details toward the conformational dynamical alterations of the enzyme as it becomes more resistant to DRV. All analyses converge to the conclusion that 9Mut-L76V takes the main responsibility for the increase of drug resistance from 8Mut to 10Mut, whereas the addition of A71V restores the catalytic efficiency of the protease for the virus to survive. The 11Mut variant, with the highest drug inhibition constant among the variants, generates the most “collapsed” conformations that effectively hinder access of both drugs and substrates to the active site. The enhanced contact-based residue community analysis summarizes multiple conformational ensembles in one community network and directly links conformational dynamics with function (i.e., inhibition constants), which can be exploited in future work as a general, efficient tool for detecting function-related dynamics and establishing potential allosteric communication pathways.

## Data Availability

Input files of MD simulations are available as attachment to the Supporting Information. MD simulations were performed with Amber16 and the Amber ff14SB force field, as described in Methods. PCA, RMSF, and distance analysis were performed with the CPPTRAJ program of Amber16. Residue-residue contact analysis and the community analysis were performed using the script files of dCNA, which are publicly available from https://github.com/The-Hamelberg-Group/dcna.

## Supporting Information Available

MD input files (ZIP)
Scree plot from the PCA of wild-type HIV-1 protease (Figure S1); modularity of HIV-1 protease relative to the number of communities (Figure S2); correlation plot between the cyan and tan communities’ residue-residue contact relative to log(*K*_i_) values (Figure S3) (PDF)
