# Supplementary material for: Evolving Mutational Buildup in HIV-1 Protease Shifts Conformational Dynamics to Gain Drug Resistance
Source: J Chem Inf Model. 2023 Jun 7;63(12):3892–902. doi: 10.1021/acs.jcim.3c00535 (PMC10302472; doi:10.1021/acs.jcim.3c00535)
Supplement: Supplementary file 2 — ci3c00535_si_002.pdf [file ci3c00535_si_002.pdf]

Supporting Information

Evolving Mutational Buildup in HIV-1 Protease Shifts Conformational Dynamics to Gain Drug Resistance

Michael Souffrant[1], Xin-Qiu Yao[1], Donald Hamelberg*[1,2]

[1]Department of Chemistry, [2]Center for Diagnostics and Therapeutics, Georgia State University, Atlanta, Georgia 30302-3965, USA.

*Corresponding Author Tel.: (404) 413-5564; E-mail: dhamelberg@gsu.edu

SI Figures

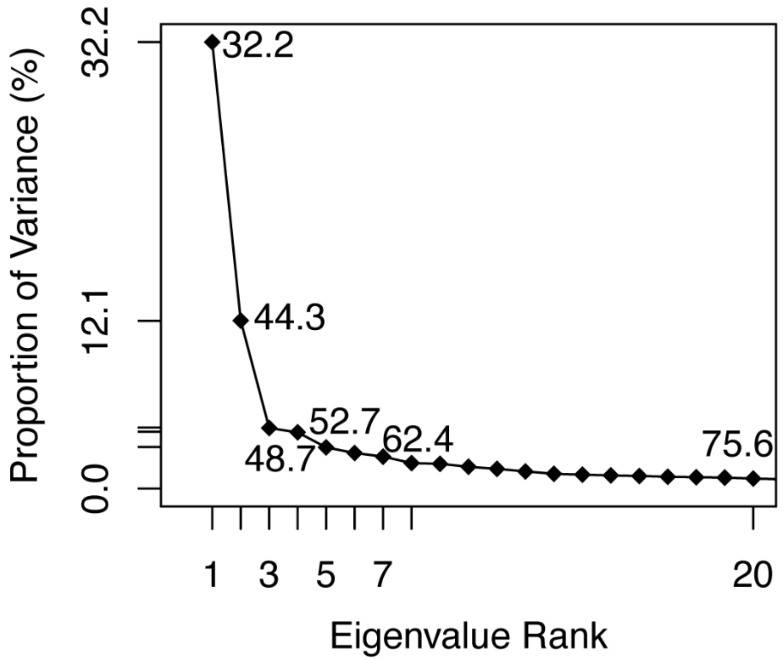

Figure S1. Scree plot from the PCA of wild-type HIV-1 protease.

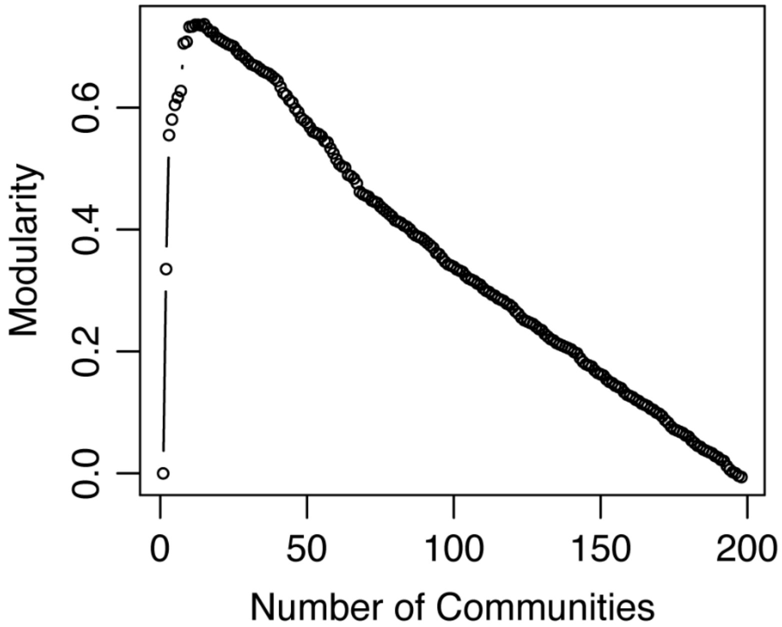

Figure S2. Modularity of HIV-1 protease relative to the number of communities.

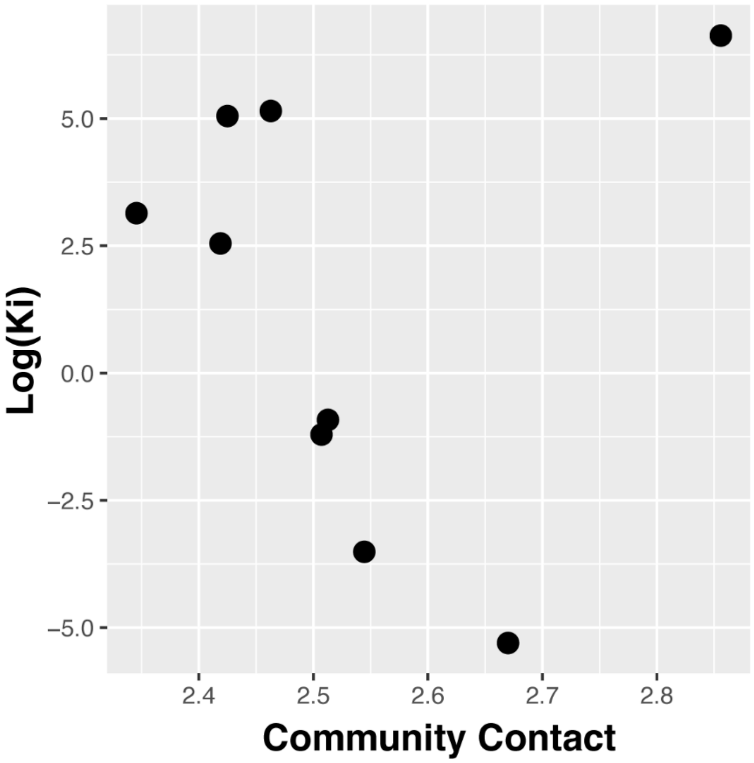

Figure S3. Correlation plot between the cyan and tan communities' residue-residue contact relative to log (K_i) values.
